# SMS text message reminders versus usual care for cervical screening attendance in London, UK: a retrospective secondary analysis of routinely collected data

**DOI:** 10.1016/j.lanprc.2026.100215

**Published:** 2026-09

**Authors:** Beth Nichol, Josephine Ruwende, Jeff Powell Davies, Kaloyan Haralampiev, Christian Von Wagner, Aikaterini Grimani, Luke Vale, Ivo Vlaev

**Affiliations:** a NIHR Policy Research Unit in Behavioural and Social Sciences, Population Health Sciences Institute, Newcastle University, Newcastle upon Tyne, UK; b School of Communities and Education, Northumbria University, Newcastle upon Tyne, UK; c Public Health, NHS England, London, UK; d Project Analytics and Insights, NHS North of England Commissioning Support Unit, John Snow House, Durham, UK; e Department of Sociology, Faculty of Philosophy, Sofia University St Kliment Ohridski, Sofia, Bulgaria; f Department of Health Services Research and Policy, London School of Hygiene & Tropical Medicine, London, UK; g Health Economics Group, Population Health Science Institute, Newcastle University, Newcastle upon Tyne, UK; h Research Department of Behavioural Science and Health, University College London, London, UK; i NIHR Policy Research Unit in Behavioural and Social Sciences, Behavioural Science Group, Warwick Business School, University of Warwick, Coventry, UK; j Centre for Behavioral and Implementation Science Interventions, Yong Loo Lin School of Medicine, National University of Singapore, Queenstown, Singapore

## Abstract

**Background:**

Previous randomised controlled trials have shown that general practitioner (GP)-endorsed text message reminders can increase cervical screening attendance. Following the implementation of these reminders across routine primary care services in London (UK), we aimed to assess their effect on likelihood of cervical screening attendance when implemented at scale.

**Methods:**

In this retrospective secondary analysis of routinely collected data, all individuals aged 24–64 years who were eligible for cervical screening within participating GP practices in London between June 21, 2021, and March 11, 2024, were included. People who had opted out of the screening programme or the text reminder service, had had a cervical screening before the initial invitation letter, or were out of the age range at the time of the initial invitation letter (ie, aged <24 years or ≥65 years) were excluded. Attendance was compared between individuals who received a GP-endorsed SMS reminder and those who did not. The main outcome was odds of cervical screening attendance within the screening cycle. Routinely collected data were obtained from the text message provider and the National Health Application and Infrastructure Services (now the UK National Health Service Cervical Screening Management System). We used binary logistic regression to examine associations between SMS reminder delivery and attendance, adjusting for previous screening behaviour, age, and deprivation. Data on sex, gender, and ethnicity were not possible to include.

**Findings:**

Of the 3 530 557 individuals assessed for eligibility, 3 127 759 were included and 402 798 were excluded. 2 112 003 (67·5%) of the 3 127 759 individuals received an SMS reminder for cervical screening and 1 015 756 (32·5%) did not. 1 091 902 (51·7%) of the 2 112 003 individuals who received an SMS reminder attended a screening, which was an increase of 1·7 percentage points since the last screening cycle (1 055 287 [50·0%] of 2 112 003), whereas 253 556 (25·0%) of 1 015 756 individuals who did not receive an SMS reminder attended a screening, which was a decrease of 2·8 percentage points since the last screening cycle (282 484 [27·8%] of 1 015 756). Successful delivery of an SMS reminder resulted in an increased likelihood of cervical screening attendance (odds ratio 3·36 [95% CI 3·31–3·40]; p<0·0001).

**Interpretation:**

The findings indicate the potential effect of SMS reminders as a scalable component of cervical screening systems while emphasising the importance of digital reach, contact-data quality, and equitable implementation. Further research should evaluate differential effects considering demographic characteristics, including ethnicity and socioeconomic status.

**Funding:**

National Institute for Health and Care Research and the National Recovery and Resilience Plan of the Republic of Bulgaria.

## Introduction

Cervical cancer is currently the only cancer that can be eliminated completely as a public health concern,^1^ a goal that has been adopted by WHO through a global strategy.^2^ Cervical cancer is well controlled by high-income countries,^1^ often attributed to cervical screening and human papillomavirus (HPV) vaccination programmes.^3^ The cervical cancer screening programme in the UK is well established and is estimated to save up to 5000 lives per year.^4^ People with a cervix and aged 24·5 years and older are invited for screening every 5 years through an invitation letter and a reminder letter (hereby referred to as a follow-up letter) 18 weeks later.^5^ Unlike breast and bowel cancer screening, cervical screening coverage in England has steadily declined since 2010.^6^ Important inequalities in uptake also exist, including lower uptake in groups of minoritised ethnicity or living in deprived areas,^7^ the youngest eligible individuals,^8^ and people born in a different country.^8^ Lower participation rates in groups of minoritised ethnicity are particularly concerning given that they are also less likely to complete their HPV vaccination series.^9^

**Research in context**

**Evidence before this study**

We systematically searched MEDLINE and PsycInfo (via Ovid) on Nov 12, 2025, for papers published since database inception (MEDLINE) or Jan 1, 1987 (PsycInfo), with the search terms “cervical screening” AND “effect∗” AND (“text message” OR “SMS”). No language restrictions were applied. Additionally, forward citation searching of relevant articles was conducted. We included quantitative studies that evaluated the effectiveness of text message reminders on increasing cervical screening attendance. The search identified 44 results, of which seven unique studies were identified as relevant. In four studies, SMS messages were one component of a multicomponent intervention, making the attribution of any effects difficult. Three other studies focused on a subsample of individuals (eg, non-responders) and none were population wide. The three fully powered studies that were available evaluated SMS messages that contained details of a prebooked cervical screening appointment, instead of requiring individuals to book screening themselves. One study conducted two randomised control trials (RCTs) that evaluated the effectiveness of different behaviourally informed text message reminders on increasing cervical screening attendance in London (UK). The intention-to-treat analysis of the second RCT identified that general practitioner (GP)-endorsed text messages were most effective in improving cervical screening attendance, compared with loss-framed, gain-framed, and social norms-framed text message reminders. These RCTs provided an estimated effect size in a controlled setting and context. However, research was needed to explore whether the observed increase in cervical screening attendance could be retained when delivered at scale at a population level.

**Added value of this study**

We found that GP-endorsed SMS reminders increased the odds of cervical screening attendance when delivered at scale in a secondary analysis of routinely collected data in London, UK, which persisted when controlling for past screening behaviour and key demographic and contextual factors. The findings indicate scalability of text message reminders without losing their meaningful impact on cervical screening attendance.

**Implications of all the available evidence**

Our results show that GP-endorsed text message reminders can increase the likelihood of cervical screening attendance, offering a potential low-cost and low-intensity effective intervention. To ensure equitable implementation of text message reminders, investment into strategies to increase the coverage of text messages by health-care services is needed.

To improve cancer screening uptake, behavioural nudges have been used to encourage screening attendance through subtle changes in the environment, which make screening attendance more salient or socially desirable without restricting individual choice.^10^ Generally, text (ie, SMS) reminders are low cost, acceptable to the public, and effective in promoting appointment attendance and medical adherence.^11^ More broadly, experimental studies of cancer screening communications have shown that descriptive social-norm information can increase screening intentions among people initially disinclined to participate, and that its effect depends on how screening uptake is communicated.^12^^,^^13^ Subtle changes in the framing of nudge text messages can affect behaviour.^14^ Particularly, SMS reminders can be optimised by labelling them as sent by the individual's general practice. General practitioners (GPs) and, by proxy, general practices, are a credible, trusted, and familiar source to the public;^15^ thus individuals are more receptive to messages when endorsed by them.^16^ This effect is particularly pronounced in individuals who ordinarily have lower participation rates for cervical screening than the general population,^8^ including those with lower socioeconomic status,^16^ an immigration background,^16^ or urban residency.^16^ Two pragmatic randomised controlled trials (RCTs) conducted by Huf and colleagues^14^ assessed the effect of such GP-endorsed reminder text message on cervical screening attendance. The first RCT identified that individuals aged 24–29 years who were sent a GP-endorsed SMS reminder were more likely to attend screening than individuals who were not sent a reminder.^14^ In the second RCT in individuals aged 30–64 years, GP-endorsed SMS messages were the only behaviourally informed SMS reminder that increased screening attendance in an intention-to-treat analysis.^14^ Based on these data, GP-endorsed messages were selected for London-wide implementation of cervical screening SMS reminders,^17^ where cervical screening coverage is the lowest of any region in England, particularly for those aged 25–49 years.^6^ 97% of practices in London signed up to the policy, and initial figures from these practices reported that attendance over the first 6 months increased by 4·8% (equivalent to 13 400 more people being screened).^17^

Although useful for informing practice, these RCTs did not examine effectiveness during routine large-scale implementation or account for key contextual moderators, such as previous screening behaviour. We aimed to address this evidence gap by using data from the London-wide implementation to assess the effect of SMS reminders on odds of cervical screening attendance in a real-world setting.

## Methods

### Study design and data sources

In this retrospective secondary analysis, we used routinely collected administrative data from the UK National Health Service (NHS) Cervical Screening Management System (CSMS), which identifies individuals due for screening, manages screening-related communications, and records test results. It is a centralised screening registry containing the individual-level demographic and administrative data required to support these functions, not a comprehensive clinical record.^18^ SMS reminders were sent as standard during the study period (June 21, 2021, to March 11, 2024), following their introduction across London on Sept 1, 2018.

All people aged 24–64 years who were due for cervical screening (every 3·5 years for individuals aged 25–49 years and every 5·5 years for individuals aged 50–64 years at the time of the study period) within the study period at any participating GP practice in London were assessed for eligibility. Eligible people who had opted out of the screening programme or the text reminder service, had had a cervical screening before the initial invitation letter, or were out of the age range at the time of the initial invitation letter (ie, aged <24 years or ≥65 years) were excluded. Individuals who had had a cervical screening before the invitation letter either recognised that they were due before receiving an invitation letter, missed their last cervical screening appointment and booked before the next screening cycle, or were sent to screening early by their GP due to having symptoms. A small number of initial invitation letters were recalled with no reason provided, so these individuals were also excluded. People who received more than one initial invitation letter within 18 weeks were also removed from analysis because validating the reason for receiving more than one letter was not possible. A person might have been included more than once if they had multiple screening cycles within the intervention period (eg, if they had a positive or ambiguous test result), with each instance being treated separately. Due to limitations of the recorded data by the CSMS, extracting data on sex, gender, and ethnicity was not possible. Additionally, due to GP records in England only including one binary administrative field that conflates sex and gender, transgender men are not automatically included in the screening invite and reminder process if registered as male with their GP practice, even if they have a cervix.

The research proposal was approved by the NHS Cervical Screening Research Innovation and Data Advisory Committee (reference CSP_0067). The inclusion of individuals did not require individual informed consent and harms were not assessed as this was a secondary analysis of routinely collected NHS data (appendix pp 1–2).^19^^,^^20^

### Procedures

Invitation letters were sent from the national screening programme. All individuals received an invitation letter (figure 1). Participating practices entered into a data sharing agreement with NHS London that enabled mobile telephone numbers of people who were eligible for screening and had not opted out from receiving SMS messages to be sent from GP clinical systems to iPLATO, a messaging system used by the NHS at the time of the study.^20^ A list of individuals who were sent an invitation letter was generated weekly by North of England Care System Support (NECS) Data Management on behalf of NHS England—London and passed to iPLATO, which then attempted to pull individuals’ mobile number from GP systems and sent SMS reminders to retrieved mobile numbers approximately 2 weeks after the invitation letter (range 10–16 days).

**Figure 1:**
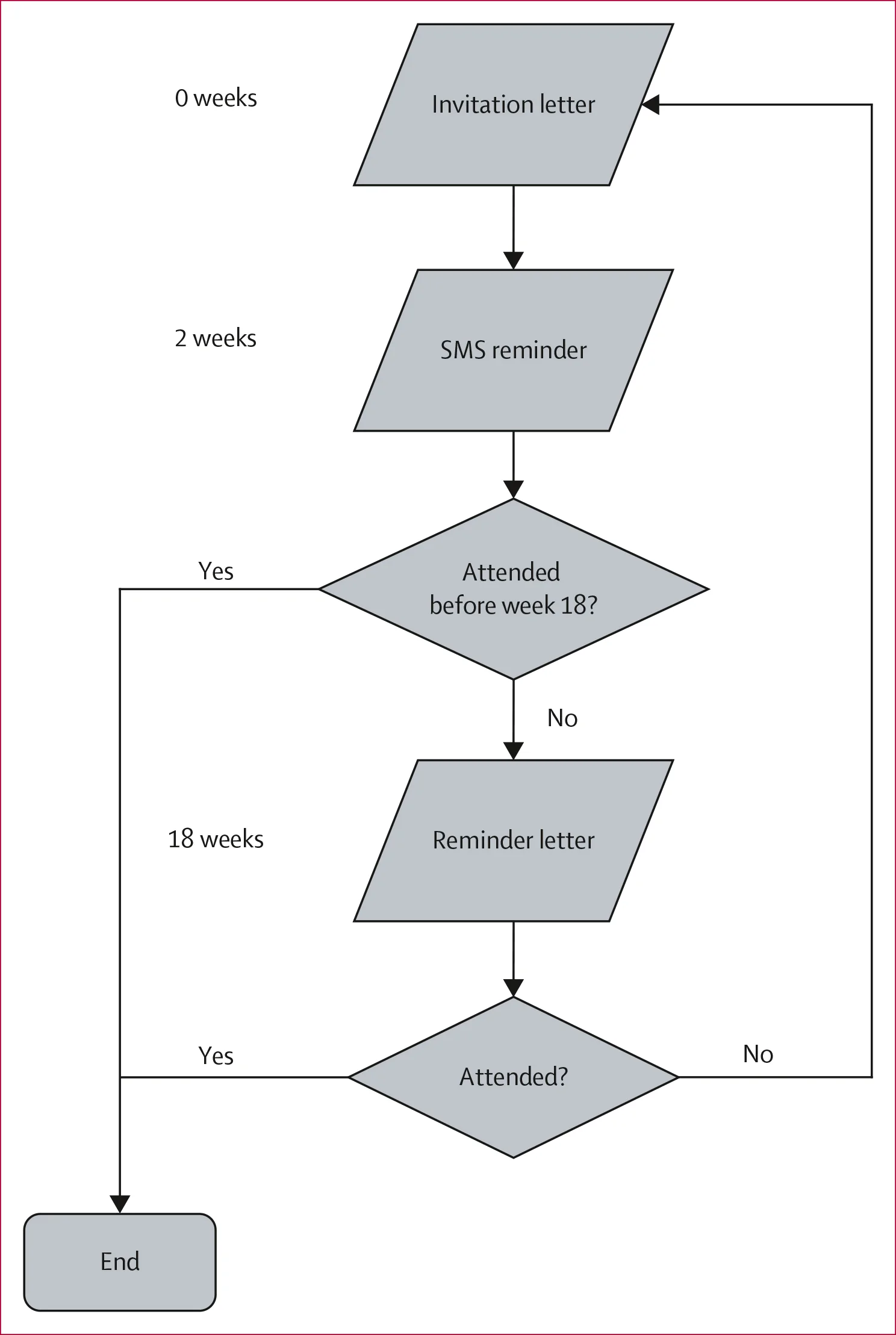
Cervical screening invitation and reminder process during the study period

The SMS text reminder contained the individual’s GP practice name, telephone number, and links to NHS information and the privacy notice. Message content was standardised but personalised by GP surgeries (with the name and telephone number of the GP practice only). Thus, reminder text read:

“<GP SURGERY NAME>: Your cervical smear test is due. To book, please call <GP phone number>. For further information, visit <NHS.UK>. To opt out of text reminders please call your GP. For NHS England privacy notice, visit <link>.”

Although the text message service was adopted by most practices, some individuals would not receive a text due to recording errors (eg, NHS number reported in the wrong form field) or system errors (eg, issues syncing updated individual data to GP practice records), or due to the actions of the individual (eg, because they opted out of receiving text messages, did not share their mobile number with their GP practice, or did not own a mobile telephone). Therefore, the differential delivery status of the SMS reminder created two comparison groups: SMS delivered and SMS not delivered. Telephone number availability and SMS delivery status for each individual (ie, opted out, delivered, or undeliverable) was sent by iPLATO to NECS. Data on invitation dates, screening attendance, demographics, and previous screening history were extracted and linked by NECS from the National Health Application and Infrastructure Services (now the CSMS).

All data were transferred to NECS. Individual-level data remained within NECS and were not shared externally. Data were anonymised before analysis. Analysis outputs and aggregate-level data were shared with the Article authors and NHS England.

Usual practice across London included a second SMS sent after the 18-week reminder letter, but this second SMS was not included in our analysis. GP practices that did not consent to the text message service followed the same process as GP practices that did consent, without the SMS reminder.

### Outcomes

The main outcome was odds of attendance of cervical screening following an initial invitation letter and before the end of the current screening cycle. The current screening cycle ended on June 21, 2024 (ie, the last available data in the series), when the next initial invitation letter was sent, or the end of the age-specific period of each screening cycle, whichever was first.

### Statistical analysis

First, a contingency table was created. Receipt of the SMS reminder was defined as a sent text message with no subsequent failure reason. The primary analysis consisted of four binary logistic regression models (hereafter referred to as models 1–4) with screening attendance as the dependent variable: SMS delivery as the independent variable (model 1), controlling for the day of the week and month the SMS reminder was sent (model 2), also controlling for previous screening attendance (model 3, grouped into: never been screened and not invited previously, never been screened although invited previously, screened in the previous cycle, and screened but not in the previous screening cycle), and adding demographic variables (model 4) of age at the point the screening invitation was sent and Index of Multiple Deprivation (IMD) decile based on the postcode to where the invitation letter was sent. The models included delivery of an SMS reminder, delivery of a follow-up letter, and the interaction between both variables.

Exploratory groupings were created post hoc based on the delivery of both reminders and the screening behaviour of individuals: (1) individuals who received an invitation letter only (the most likely reason being that the person attended screening within 18 weeks and an SMS message reminder was not delivered to them), (2) individuals who received an SMS reminder after the invitation letter (individuals from GP practices enrolled onto the text message service who attended screening within 18 weeks of invitation and thus were not sent a follow-up letter), (3) individuals who received a follow-up letter after the invitation letter (including individuals from GP practices that did not participate in the text message service, although the most likely reason is that the SMS message reminder was undeliverable and the individual did not attend screening within the screening cycle after being invited), and (4) individuals who received both an SMS reminder and a follow-up letter after an invitation letter (individuals from GP practices enrolled onto the text message service who had not attended screening within 18 weeks of being invited).

Individuals with incomplete data were removed from analyses. Across all models, each categorical variable was one-hot-encoded, transforming each category into a binary indicator variable (1 for the observed category and 0 otherwise), leaving no missing values. The unit for IMD decile category was one tenth. All analyses were conducted using Python (version 3.12.7) and R (version 4.4.3). Log odds ratios were converted to odds ratios (ORs) to assist with interpretation.

### Role of the funding source

The funders of the study had no role in study design, data collection, data analysis, data interpretation, or writing of the report.

## Results

Of the 3 530 557 individuals assessed for eligibility, 3 127 759 were included and 402 798 were excluded (figure 2). Of the 3 127 759 initial invitation letters sent, no attempt was made to send an SMS to 519 276 individuals (figure 2). Text messages failed to deliver primarily because the carrier did not recognise the telephone number of the individual (ie, message failed) or because the mobile telephone was switched off for longer than 72 h after the delivery attempt (ie, message expired). The frequency of the individual reasons why an SMS reminder could not be scheduled was not possible to ascertain, although the main reason was that there was insufficient detail to match the individual to a GP practice (eg, no valid NHS number; figure 2). For 116 243 (22·4%) of the 519 276 individuals for whom no SMS message was attempted, an SMS message was not sent because their GP practice did not participate in the SMS message service. For the remaining 496 480 individuals who did not receive an SMS, their details were sent to iPLATO but the SMS message was undeliverable. 2 112 003 (67·5%) of the 3 127 759 individuals who were sent an invitation letter received SMS reminders and 1 015 756 (32·5%) did not. 2 061 315 (97·6%) SMS messages were sent on a Wednesday, with the remainder sent on a Tuesday (16 896 [0·8%]) or a Thursday (33 792 [1·6%]).

**Figure 2:**
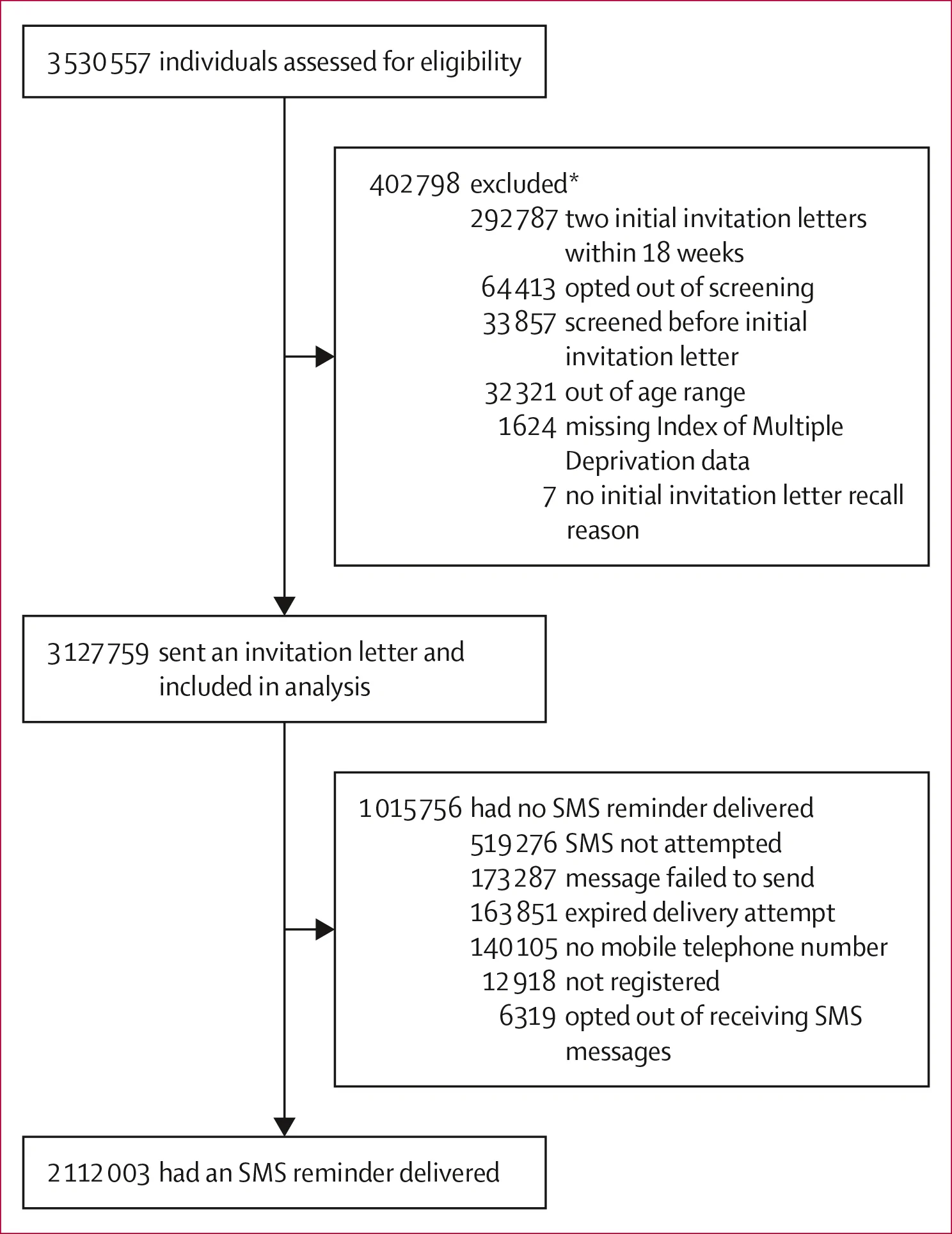
Study profile ∗Some individuals had multiple reasons for exclusion, so these categories are not mutually exclusive.

Individuals who received an SMS reminder were more likely to have been screened previously than those who did not receive a reminder (table 1). More individuals who received an SMS reminder had never been screened before, compared with those who did not receive a reminder (table 1).

**Table 1:** Available baseline characteristics

	No SMS delivered (n=1 015 756)	SMS delivered (n=2 112 003)
Age, years	36·6 (9·7)	38 4 (10·5)
Index of Multiple Deprivation decile		
1 (ie, most deprived)	23 293 (2·3%)	43 803 (2·1%)
2	140 934 (13·9%)	318 712 (15·1%)
3	192 856 (19·0%)	398 504 (18·9%)
4	164 003 (16·1%)	321 725 (15·2%)
5	121 254 (11·9%)	259 218 (12·3%)
6	115 477 (11·4%)	230 727 (10·9%)
7	86 555 (8·5%)	175 504 (8·3%)
8	80 033 (7·9%)	153 966 (7·3%)
9	61 306 (6·0%)	139 037 (6·6%)
10 (ie, least deprived)	30 045 (3·0%)	70 807 (3·4%)
Previous screening behaviour		
Never been screened (first invitation)	190 227 (18·7%)	331 413 (15·7%)
Never been screened (invited previously)	276 486 (27·2%)	320 840 (15·2%)
Screened, but not in the previous screening cycle	266 559 (26·2%)	404 463 (19·2%)
Screened in the previous screening cycle	282 484 (27·8%)	1 055 287 (50·0%)

Data are mean (SD) or n (%).

3 127 759 individuals with contact with the screening process (representing 2 680 121 unique individuals) were included in the analysis. 1 091 902 (51·7%) of the 2 112 003 individuals who received an SMS reminder attended a cervical screening, which was an increase of 1·7 percentage points since the last screening cycle (1 055 287 [50·0%] of 2 112 003). In comparison, 253 556 (25·0%) of 1 015 756 individuals who did not receive an SMS reminder attended a cervical screening, which was a decrease of 2·8 percentage points since the last screening cycle (282 484 [27·8%] of 1 015 756; appendix p 3).

With all covariates included (model 4), successful delivery of an SMS reminder resulted in an increased likelihood of cervical screening attendance (OR 3·36 [95% CI 3·31–3·40]; p<0·0001; table 2).

**Table 2:** Binary logistic regression models 1–4

	Model 1	Model 2	Model 3	Model 4
SMS delivery				
No successful delivery of text message reminder	1 (ref)	1 (ref)	1 (ref)	1 (ref)
Successful delivery of text message reminder	3·31 (3·27–3·35)	4·29 (4·23–4·34)	3·36 (3·31–3·40)	3·36 (3·31–3·40)
Follow-up letter				
Not sent	1 (ref)	1 (ref)	1 (ref)	1 (ref)
Sent	0·06 (0·06–0·07)	0·06 (0·06–0·06)	0·08 (0·08–0·08)	0·08 (0·08–0·08)
Interaction between successful delivery of text message reminder and follow-up letter sent	0·77 (0·75–0·78)	0·68 (0·67–0·69)	0·67 (0·66–0·68)	0·67 (0·66–0·68)
Day of the week SMS was sent				
Tuesday	NA	1·26 (1·22–1·31)	1·34 (1·29–1·39)	1·34 (1·29–1·39)
Wednesday	NA	1 (ref)	1 (ref)	1 (ref)
Thursday	NA	1·18 (1·15–1·21)	1·27 (1·23–1·30)	1·27 (1·23–1·30)
Month of the year SMS was sent				
January	NA	1 (ref)	1 (ref)	1 (ref)
February	NA	0·56 (0·55–0·57)	0·56 (0·55–0·57)	0·56 (0·55–0·57)
March	NA	0·24 (0·24–0·25)	0·31 (0·30–0·31)	0·31 (0·30–0·31)
April	NA	0·79 (0·77–0·80)	1·01 (0·99–1·02)	1·01 (0·99–1·02)
May	NA	0·75 (0·73–0·76)	0·95 (0·94–0·97)	0·95 (0·94–0·97)
June	NA	0·84 (0·83–0·85)	0·97 (0·95–0·98)	0·97 (0·95–0·98)
July	NA	0·89 (0·88–0·90)	1·03 (1·02–1·05)	1·03 (1·02–1·05)
August	NA	0·87 (0·86–0·88)	0·99 (0·98–1·01)	0·99 (0·98–1·01)
September	NA	0·87 (0·86–0·88)	0·95 (0·94–0·97)	0·95 (0·94–0·97)
October	NA	0·87 (0·86–0·89)	0·93 (0·92–0·95)	0·93 (0·92–0·95)
November	NA	0·73 (0·72–0·74)	0·81 (0·80–0·82)	0·81 (0·80–0·82)
December	NA	0·70 (0·70–0·71)	0·81 (0·79–0·82)	0·81 (0·80–0·82)
Previous screening participation				
Never been screened (first invitation)	NA	NA	1 (ref)	1 (ref)
Never been screened (invited previously)	NA	NA	0·41 (0·40–0·41)	0·41 (0·41–0·42)
Screened, but not in the previous screening cycle	NA	NA	0·72 (0·72–0·73)	0·74 (0·74–0·75)
Screened in the previous screening cycle	NA	NA	3·30 (3·27–3·33)	3·37 (3·34–3·40)
Age when the cervical cancer screening invitation letter was sent	NA	NA	NA	1·00 (1·00–1·00)
Index of Multiple Deprivation decile of postcode to which the cervical cancer screening invitation letter was sent	NA	NA	NA	1·02 (1·02–1·02)

Data are odds ratio (95% CI). N=3 127 759 for all models. See the appendix (p 4) for the sample size and cervical screening attendance for the reference categories. Model 1: SMS delivery as the independent variable. Model 2: controlling for the day of the week and month the SMS reminder was sent. Model 3: also controlling for previous screening attendance grouped into: never been screened and not invited previously, never been screened although invited previously, screened in the previous cycle, and screened but not in the previous screening cycle. Model 4: adding demographic variables of age at the point the screening invitation was sent and Index of Multiple Deprivation decile based on the postcode to where the invitation letter was sent. NA=not applicable.

Exploratory groupings were: received neither reminder (196 729 [6·3%] of 3 127 759), received the text reminder only (798 637 [25·5%]), received the letter reminder only (819 027 [26·2%]), or received both (1 313 366 [42·0%]; table 3).

**Table 3:** Binary logistic regression models 1–4 assessing exploratory groupings

	Model 1	Model 2	Model 3	Model 4
Exploratory group				
Both	1 (ref)	1 (ref)	1 (ref)	1 (ref)
SMS only	20·16 (19·99–20·31)	23·92 (23·71–24·12)	18·41 (18·25–18·58)	18·36 (18·19–18·52)
Follow-up letter only	0·39 (0·39–0·40)	0·34 (0·34–0·35)	0·45 (0·44–0·45)	0·45 (0·44–0·45)
Neither	6·09 (6·03–6·15)	5·57 (5·51–5·64)	5·49 (5·42–5·56)	5·47 (5·40–5·54)

Data are odds ratio (95% CI). N=3 127 759 for all models. See the appendix (p 4) for the sample size and cervical screening attendance for the reference categories. Model 1: SMS delivery as the independent variable. Model 2: controlling for the day of the week and month the SMS reminder was sent. Model 3: also controlling for previous screening attendance grouped into: never been screened and not invited previously, never been screened although invited previously, screened in the previous cycle, and screened but not in the previous screening cycle. Model 4: adding demographic variables of age at the point the screening invitation was sent and Index of Multiple Deprivation decile based on the postcode to where the invitation letter was sent.

## Discussion

GP-endorsed SMS reminders increased odds of cervical screening attendance when deployed at scale across a heterogeneous urban population in London, UK, in line with findings from previous RCTs.^14^ This finding addresses a key concern in behavioural science regarding whether intervention effects observed under controlled conditions attenuate in real-world settings. Because the data source was limited to the CSMS, which only collects data relevant to its function, there is a likelihood that there were important differences in the baseline characteristics of the two groups (SMS delivered and SMS not delivered) that were not accounted for in the analyses. Therefore, the observed effects must be interpreted cautiously. Groups were assigned based on the delivery of an SMS reminder rather than whether participating GP practices enrolled onto the text message service, likely overestimating the real-world effectiveness of implementing reminder text messages. The effectiveness of text message reminders on likelihood of cervical screening attendance emphasises the need to adopt strategies to increase the coverage of text messages by health-care services. An important caveat to the findings is that some of the analyses (eg, the delivery of an invitation letter and exploratory groupings) depended on the response pattern of individuals, limiting the conclusions that can be drawn from them.

Text message reminders did not mitigate the negative relationship between deprivation and likelihood of cervical screening attendance in our analysis or previously**,**^14^ indicating that text message reminders should be used in conjunction with interventions that address the wider determinants (eg, structural and economic) causes of inequalities in cervical screening attendance. Relatedly, the reduced likelihood of screening attendance for individuals who had never been screened before was not attenuated by SMS delivery, identifying a population who additionally require more intensive outreach to address specific barriers to attendance.

Regarding developments in the UK cervical screening programme, our findings are important to consider. First, at-home self-sampling is an emerging intervention that is becoming increasingly available to increase cervical screening attendance,^8^ although increased uptake has only been observed in opt-out models and not opt-in models.^21^ Self-sampling likely helps to address different barriers to cervical screening than text message reminders, such as fear and embarrassment.^22^ Behaviourally informed reminders increased the return of HIV self-sampling kits in England,^23^ indicating that text message reminders could be used in conjunction with self-sampling to increase cervical screening uptake further. Also, the switch from initial cytology screening (ie, the Pap test) to high-risk HPV testing as of Dec 1, 2019, in the UK^5^ increased testing sensitivity,^8^ identifying individuals who are at higher risk of cervical cancer (ie, HPV positive) and inviting them for more frequent cervical screening. Reminder text messages could be tailored for people who are high-risk HPV positive, which could increase the effectiveness of SMS reminders for this group. Tailored messaging based on risk increased the intention to attend colorectal cancer screening compared with generalised information about risk.^24^ Given that a previous RCT, which tested differing content of the reminder text messages, identified that loss-framed messages (ie, that failing to attend screening results in X identifiable deaths each year) were most effective in increasing cervical screening attendance for those who received a reminder,^14^ loss-framed messages could be tailored for those who are HPV positive to maximise their effect.

Finally, this nudge intervention assumed that cervical screening attendance was the desired outcome. In reality, attendance carries benefits and harms, and individuals must be supported to reach an informed choice. Previous experimental work suggests that behaviourally informed screening messages need not necessarily undermine information seeking or comprehension, although these outcomes should be considered explicitly when designing nudges.^13^ As such, implementing nudge strategies alongside, not in lieu of, more intensive interventions that facilitate informed decision making is important, particularly those targeted at underserved populations. Aligning nudges with changing national guidelines is also essential; thus, continual review is required. For example, although cervical screening is still recommended for people who are HPV vaccinated in the UK (implemented from 2008 for children aged 12–13 years), guidelines are expected to evolve with emerging evidence on the effectiveness of vaccination.^25^ Since this analysis was conducted, cervical screening invitations in England are also sent by the NHS app, followed by an SMS if the app notification is not opened.^26^ Invitations and reminders sent via the NHS app could allow the potential of bidirectional communication,^27^ further streamlining the process of booking an appointment.

The main caveat of this analysis is that assessment of effectiveness was based on text message delivery. Previous RCTs identified that only 31·1–41·2% of texts were successfully delivered, depending on age subgroups and IMD decile, with a lower proportion of texts being successfully delivered with increasing age and increasing deprivation.^14^ Another key limitation is that, although the multiple logistic regression models we used assumed independence of observations, 3 127 759 individuals with contact with the screening process were included in the current analysis, representing 2 680 121 unique individuals. Consequently, any within-person correlation was not accounted for and SEs might be underestimated. However, the effect of this multiplicity is arguably small given the relatively low number of repeat observations and the large sample size. We were unable to attain individual reasons for why an SMS could not be scheduled, which made up over half of the undelivered SMS messages. Thus, the comparability across groups and generalisability of the findings to the general population is uncertain. Additionally, we could not account for other important factors that were not included in CSMS records, such as ethnicity and clustering of GP practices, which could account for relevant potential predictors such as the delivery of person-centred care. The clustering of GP practices is particularly important information given that registration of the GP practice with the text message service partly determined SMS delivery. A full economic analysis was not conducted, which would have examined cost-effectiveness and would have ideally considered the downstream effects on cervical cancer incidence by grade, recurrences, and mortality. Although text message reminders have been implemented nationally for the UK cervical screening programme, a cost-effectiveness analysis would inform the implementation of text message reminders for other screening programmes. A cost-effectiveness analysis could also explore any unintended negative effects of text message reminders. For example, in this analysis, data were not available to explore whether SMS reminders increased the number of individuals who opted out of receiving SMS messages from their GP practice.

Text message reminders to encourage breast and colorectal cancer screening have been shown to be effective and cost-effective.^28^^,^^29^ A consensus study that included experts in UK-based screening programmes agreed that GP-endorsed text message reminders were an evidence-based and feasible strategy that should be incorporated across screening programmes.^27^ The UK is one of several countries with a central registry of cervical screening records, facilitating the implementation of text message reminders at scale.^25^ This implementation in countries without a central registry might be impeded, relying on local delivery through primary care services. Thus, exploring implementation in these settings is important.

A key priority for both research and general practice is to maximise delivery coverage of SMS reminders. The effectiveness of such interventions is contingent on successful delivery, and incomplete contact information for individuals or gaps in digital access could limit the population effect. Future work should focus on improving equitable delivery of text message reminders and understanding how intervention effects vary across demographic groups, particularly by ethnicity and socioeconomic status, to ensure equitable implementation at scale.

## Data sharing

Individual-level data remained within NECS and were not obtained for this study. Only analysis outputs and aggregate-level data were shared with the study authors that were not involved in data analysis and NHS England. The Python code used for data analysis is publicly available on GitHub (https://github.com/BethNicholNewcastle/Cervical-screening-reminders).

## Declaration of interests

CVW receives funding from the NIHR Policy Research Unit in Cancer Awareness, Screening and Early Diagnosis. All other authors declare no competing interests.
