# Supplementary material for: SMS text message reminders versus usual care for cervical screening attendance in London, UK: a retrospective secondary analysis of routinely collected data
Source: Lancet Prim Care. 2026 Sep;2(9):None. doi: 10.1016/j.lanprc.2026.100215 (PMC13662795; doi:10.1016/j.lanprc.2026.100215)
Supplement: Supplementary file 1 [file mmc1.pdf]

THE LANCET
Primary Care

Supplementary appendix

This appendix formed part of the original submission and has been peer reviewed. We post it as supplied by the authors.

Supplement to: Nichol B, Ruwende J, Powell Davies J, et al. SMS text message reminders versus usual care for cervical screening attendance in London, UK: a retrospective secondary analysis of routinely collected data. *Lancet Prim Care* 2026. https://doi.org/10.1016/j.lanprc.2026.100215

Contents

Appendix 1: Completed STROBE checklist for case-control studies. 1
Appendix 2: Contingency table for the primary analysis. 3
Appendix 3: Reference categories for the binary logistic regressions. 4

Appendix 1: Completed STROBE checklist for case-control studies.

F = Figure. SM = Supplementary Material.

	Item No	Recommendation	Page No
**Title and abstract**	1	(*a*) Indicate the study's design with a commonly used term in the title or the abstract	1
		(*b*) Provide in the abstract an informative and balanced summary of what was done and what was found	2
**Introduction**			
Background/rationale	2	Explain the scientific background and rationale for the investigation being reported	4-5
Objectives	3	State specific objectives, including any prespecified hypotheses	5
**Methods**			
Study design	4	Present key elements of study design early in the paper	5
Setting	5	Describe the setting, locations, and relevant dates, including periods of recruitment, exposure, follow-up, and data collection	6-8
Participants	6	(*a*) Give the eligibility criteria, and the sources and methods of case ascertainment and control selection. Give the rationale for the choice of cases and controls	7
		(*b*) For matched studies, give matching criteria and the number of controls per case	7
Variables	7	Clearly define all outcomes, exposures, predictors, potential confounders, and effect modifiers. Give diagnostic criteria, if applicable	8-9
Data sources/ measurement	8*	For each variable of interest, give sources of data and details of methods of assessment (measurement). Describe comparability of assessment methods if there is more than one group	8-9
Bias	9	Describe any efforts to address potential sources of bias	N/A
Study size	10	Explain how the study size was arrived at	7
Quantitative variables	11	Explain how quantitative variables were handled in the analyses. If applicable, describe which groupings were chosen and why	9-10
Statistical methods	12	(*a*) Describe all statistical methods, including those used to control for confounding	9-10
		(*b*) Describe any methods used to examine subgroups and interactions	9-10
		(*c*) Explain how missing data were addressed	10
		(*d*) If applicable, explain how matching of cases and controls was addressed	N/A
		(*e*) Describe any sensitivity analyses	N/A
**Results**			

Participants	13*	(a) Report numbers of individuals at each stage of study—eg numbers potentially eligible, examined for eligibility, confirmed eligible, included in the study, completing follow-up, and analysed	F2
		(b) Give reasons for non-participation at each stage	F2
		(c) Consider use of a flow diagram	F2
Descriptive data	14*	(a) Give characteristics of study participants (eg demographic, clinical, social) and information on exposures and potential confounders	T1
		(b) Indicate number of participants with missing data for each variable of interest	SM
Outcome data	15*	Report numbers in each exposure category, or summary measures of exposure	F2

Main results	16	(*a*) Give unadjusted estimates and, if applicable, confounder-adjusted estimates and their precision (eg, 95% confidence interval). Make clear which confounders were adjusted for and why they were included	T1, T2, 9
		(*b*) Report category boundaries when continuous variables were categorized	N/A
		(*c*) If relevant, consider translating estimates of relative risk into absolute risk for a meaningful time period	N/A
Other analyses	17	Report other analyses done—eg analyses of subgroups and interactions, and sensitivity analyses	T2
**Discussion**			
Key results	18	Summarise key results with reference to study objectives	13-14
Limitations	19	Discuss limitations of the study, taking into account sources of potential bias or imprecision. Discuss both direction and magnitude of any potential bias	15
Interpretation	20	Give a cautious overall interpretation of results considering objectives, limitations, multiplicity of analyses, results from similar studies, and other relevant evidence	15
Generalisability	21	Discuss the generalisability (external validity) of the study results	14-15
**Other information**			
Funding	22	Give the source of funding and the role of the funders for the present study and, if applicable, for the original study on which the present article is based	16

Appendix 2: Contingency table for the primary analysis.

	**Received SMS**	**Did not receive SMS**
**Not screened**	1,020,101	762,200
**Screened**	1,091,902	253,556
**Total**	**2,112,003**	**1,015,756**

Appendix 3: Reference categories for the binary logistic regressions.

Reference Category	Number who attended screening	Total in reference category	% attended screening
No successful delivery of text messaging reminder (reference)	253,556	1,015,756	25.0%
Follow-up letter not sent (reference)	852,058	995,366	85.6%
SMS sent on Wednesday	1,141,634	2,568,815	44.4%
SMS sent in January	134,141	297,565	45.1%
Never been screened (first invitation)	162,836	521,640	31.2%
Intervention group: received both SMS and follow-up letter	380,014	1,313,366	28.9%
